# Melatonin implantation improved the egg-laying rate and quality in hens past their peak egg-laying age

**DOI:** 10.1038/srep39799

**Published:** 2016-12-23

**Authors:** Yaxiong Jia, Minghui Yang, Kuanfeng Zhu, Liang Wang, Yukun Song, Jing Wang, Wenxiang Qin, Zhiyuan Xu, Yu Chen, Guoshi Liu

**Affiliations:** 1 Beijing Animal Husbandry Station, Beijing, China; 2 National Engineering Laboratory for Animal Breeding, Key Laboratory of Animal Genetics and Breeding of the Ministry of Agriculture, Beijing Key Laboratory for Animal Genetic Improvement, College of Animal Science and Technology, China Agricultural University, Beijing, China; 3 College of Animal Science and Technology, Jilin Agricultural University, Changchun, Jilin, China

## Abstract

The egg-laying rates of hens approximately 470 days of age exhibited a positive correlation to blood melatonin levels. The hens with an egg-laying rate <30%, 30~90% and ≥90% had blood melatonin levels of 5.8 ± 2.6, 74.0 ± 32.9 and 445.9 ± 115.3 ng/ml, respectively. When 10 mg of melatonin was implanted into the hens at 300, 360, 470 and 550 days of age, the egg-laying rates increased 4.63 ± 0.46%, 8.38 ± 1.45%, 4.93 ± 0.85% and 7.93 ± 0.91%, respectively, compared to that of the controls. Melatonin implantation in hens at 300–470 days of age was observed to enhance egg production and reduce the rate of appearance of sharpei eggs. Melatonin (10 mg) implanted in hens 360 days of age did not influence the blood levels of progesterone (P4) or the gene expression levels of ovarian follicle stimulating hormone receptor *(FSHR),* luteinizing hormone receptor *(LHR), oestradiol* receptor alpha *(ERα),* superoxide dismutase 2 *(SOD2)* or melatonin receptor 1 *(MT1).* In contrast, melatonin significantly elevated the serum oestradiol-17β (E2) content, down-regulated the gene expression of gonadotropin-inhibitory hormone receptor *(GnIHR)*, and enhanced the expression of melatonin receptor 2 *(MT2)*. This result indicates that the improved egg-laying rate by melatonin was the result of increased serum oestradiol and decreased ovarian *GnIHR*. These alterations may be mediated by *MT2* activation.

Several studies have reported that melatonin is capable of regulating the reproductive activities of birds. For example, in jungle bush quail (*Perdicula asiatica)*, the activity of their pineal glands exhibited an inversed relationship with their ovarian performance^1^. Melatonin administration for 30 days completely suppressed the seasonal gonadal growth in male Indian finches (*Estrilda amandava*)^2^. For the white leghorn roosters, melatonin treatment suppressed their LH secretion in a dose- and time-related manner^3^. Melatonin could also up-regulate the expression of gonadotropin-inhibitory hormone in the avian brain and thus suppressed pituitary gonadotropin secretion^4^.

Many recent studies, however, reported beneficial effects of melatonin on animal reproduction. Melatonin application promoted the maturation and development of oocytes as well as early embryos in mammals including the mouse^5^, human^6^, porcine^7^, bovine^8^ and sheep^9^. In addition to mammals, melatonin has also been shown to have beneficial effects on birds. For example, a melatonin supplement could improve the feeding efficiency of chickens and promote their growth^10^,^11^. In birds, melatonin also functions as an immunoenhancement agent^12^. Its supplementation elevated cellular and humoral immune responses in Japanese quail^13^ and chicken^14^,^15^,^16^. The signalling pathway analysis showed that both cellular and humoral immunoresponses triggered by melatonin were exclusively mediated by its receptor subtype MT2 (Mel 1b)^17^.

Egg production is a complex process that not only involves the reproductive system but also depends on the availability of specific nutrients and the efficiency of their utilization. For example, melatonin, as a nutrient, may enhance the egg laying productivity of hens. The egg-laying peak is a period when the egg-laying rate is higher than 90% in hens and often appears in hens before the age of 300 days^18^,^19^. After that period, their egg-laying productivity declines with age^20^. Previous melatonin research has focused on the period around the egg-laying peak or earlier in chickens^3^,^11^,^16^,^21^,^22^. There is no report related to the effects of melatonin on hens that are past their egg-laying peak. In the current study, the effects of melatonin on the egg production of these hens will be investigated.

## Results

### Higher blood melatonin levels in hens 470 days of age were associated with more egg production

**Figure 1:**
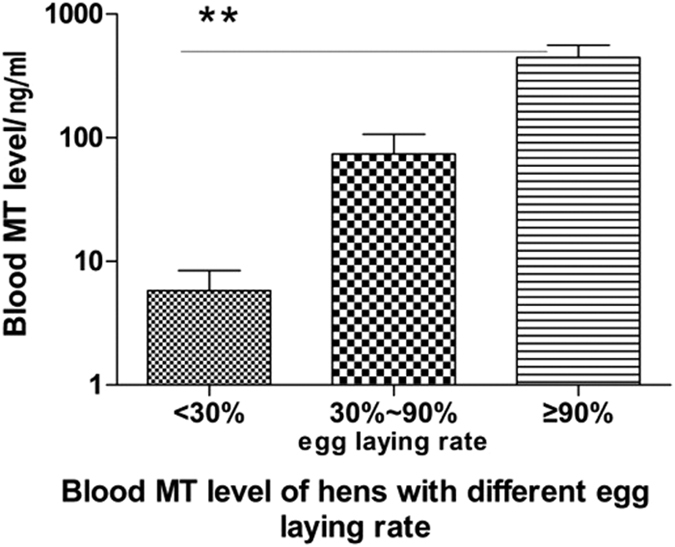
The association of blood melatonin levels and egg-laying rates in hens (440–470 days old). Egg laying numbers were recorded daily for a month before melatonin implantation. The blood was collected from the different groups classified by the egg-laying rates (n = 12). Data were expressed as the means ± SEM. “**” represents extremely significant (p < 0.01).

The results are listed in Fig. 1. It was shown that hens 470 days of age with an egg-laying rate more than 90% had significantly higher melatonin levels than those with an egg-laying rate below 30% (445.9 ± 115.3 vs 5.8 ± 2.6 ng/ml, respectively).

### Effect of melatonin implantation on the egg-laying rate

**Figure 2:**
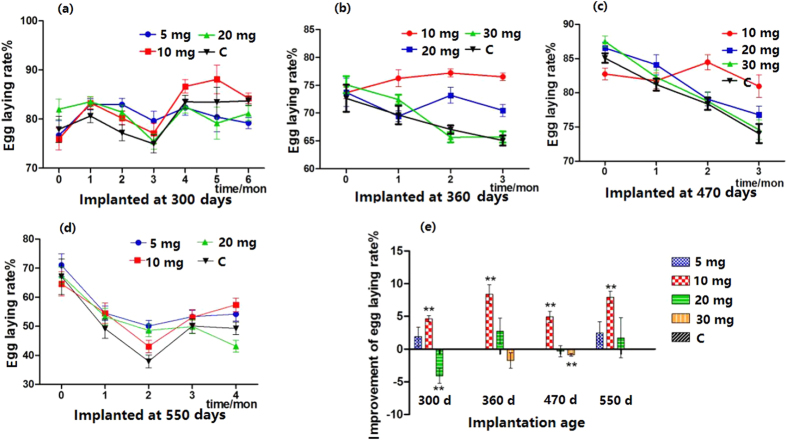
Effect of melatonin implantation on egg-laying rate. (**a–d**) Show the egg-laying rate of hens at different ages after melatonin implantation. (**e**) Shows the summary of the effects of melatonin implantation on the egg-laying rate in hens at different ages. “**” represents extremely significant (p < 0.01).

When melatonin (10 mg) was implanted into hens 360 days of age, their egg-laying rates significantly increased compared to those of their untreated counterparts, and this increase lasted for at least 6 months; however, the effects of implantation of 5 mg of melatonin lasted only 3 months (Fig. 2a). Similar effects were observed in the hens 470 and 550 days old (Fig. 2b–d). The results of melatonin (10 mg) treatment were more significant in the hens 550 days old. Throughout the experimental period, the egg-laying rate of old hens with melatonin implantation was significantly higher than those of their untreated counterparts (Fig. 2d). The most effective dose of melatonin implantation was 10 mg/hen, and at this dose, the average egg-laying rate in hens of different ages (from 300–550 days) was uniformly increased at a range of 4.63–8.38% (Fig. 2e). High melatonin levels, for example 20 mg/hen, resulted in a slightly inhibitory effect on the egg-laying rate.

### Effects of melatonin implantation on total egg weight

**Figure 3:**
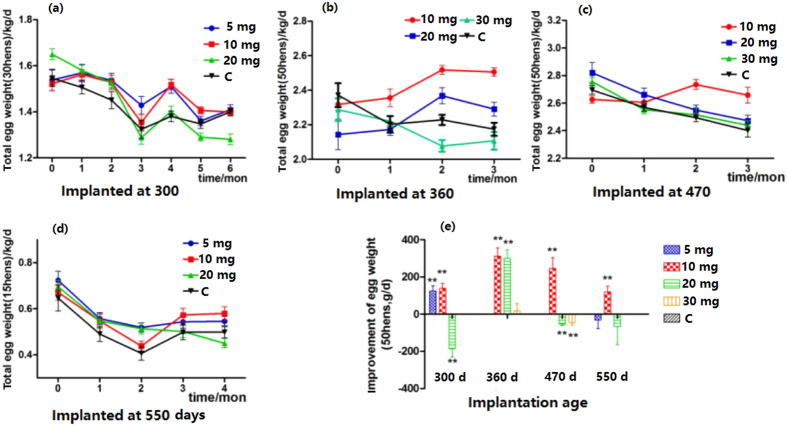
Effect of melatonin implantation on total egg weight. (**a–d**) Shows the total weight of eggs laid by hens at different ages after melatonin implantation. (**e**) Shows the summary of the effects of melatonin implantation on total egg weight (calculated daily). “**” represents significant differences of p < 0.01.

The effects of melatonin on total egg weight were variable depending on the dosages of melatonin used and the age of the hens. The results are listed in Fig. 3. In hens 300 days of age with either 5 or 10 mg of implanted melatonin, the total egg weights were significantly increased compared to the controls after 4 months of treatment (Fig. 3a). In hens 360 days of age, 20 mg of implanted melatonin increased the total egg weight compared to the controls, but 30 mg had the opposite effect (Fig. 3b). The best results were obtained from the hens 470 days of age with 10 mg of implanted melatonin. In this group, the total egg weights were significantly increased on the second month after melatonin application and maintained the highest level among all groups until the end of the study (Fig. 3c). In hens 550 days of age, different doses of implanted melatonin collectively increased their total egg weights at different time points; otherwise, the egg weights were similar to that of the control group (Fig. 3d).

Generally, melatonin implantation at a dosage of 10 mg in the hens 300–470 days old increased their egg weight 3–6 g/hen/day compared to that of their untreated counterparts. This increase lasted at least 3 months and was statistically significant (p < 0.01). However, a high dose of melatonin, for example 20 mg, had the opposite effect (Fig. 3e).

### Effects of melatonin implantation on the rates of broken and sharpei eggs

**Figure 4:**
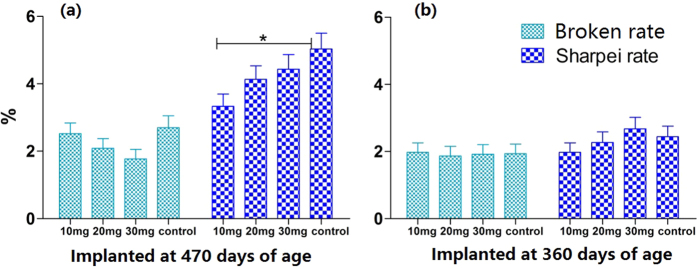
Effect of melatonin implantation on egg quality. (**a**) Shows the effect of melatonin implanted for 3 months at 470 days of age on egg broken rate and Sharpei rate. (**b**) Shows the effect of melatonin implanted for 3 months at 360 days of age on egg broken rate and Sharpei rate. Data were expressed as the mean (after 3 months of melatonin treatment) ± SEM. “*” represents significant differences (p < 0.05).

Melatonin implantation had no significant effect on the broken egg rate among the different age groups. The egg sharpei rate in hens 360 days of age implanted with 10 mg of melatonin was slightly lower than that in the control group. However, the egg sharpei rate in hens 470 days of age implanted with 10 mg of melatonin was significantly lower than that of the control (3.3 ± 0.36% vs 5.0 ± 0.47%) (p < 0.05) (Fig. 4a).

### Effects of melatonin implantation on blood E2 and P4 levels

Melatonin implantation at a dose of 10 mg significantly increased the blood level of E2 in hens 360 days of age compared to that in the controls (900.1 ± 34.4 vs 780.0 ± 44.6 pg/ml) (p < 0.05) (Table 1) and had no significant influence on other groups. Melatonin application did not impact the blood P4 levels in any groups.

### Effect of melatonin implantation on ovarian gene expression

**Figure 5:**
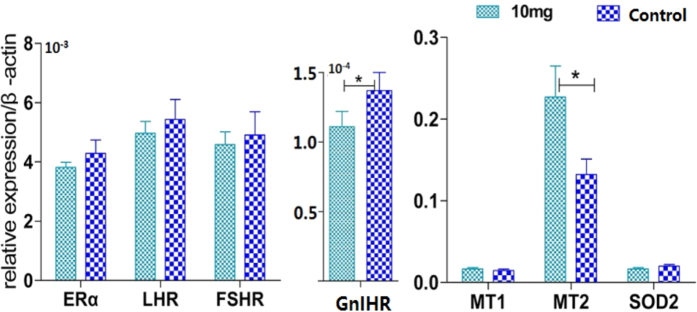
Effect of melatonin implantation on ovary gene expression. The small white follicles were used to analyse the expression of genes that are related to the effects of melatonin on egg laying. The samples were collected 3 months after melatonin implantation in hens 360 days old. Data were expressed as the mean ± SEM. “*” represents significant differences (p < 0.05).

After 3 months of melatonin implantation (10 mg), the expression of *MT2* mRNA in small white follicles of hens 360 days old was up-regulated; however, *GnIHR* expression was down-regulated. Melatonin application had no significant influence on the gene expression levels of *MT1, SOD2, ERα, FSHR* and *LHR* (Fig. 5).

## Discussion

There are few reports that are related to the effects of melatonin application on the yield of egg production in hens, especially in hens that are past the duration of their peak egg-laying rate, such as in the hens 470 to 550 days old. In the current study, it was found that in hens of 470 days of age, the egg-laying rate was positively associated with blood melatonin levels. Furthermore, melatonin implantation at a dose of 10 mg significantly improved their egg-laying rate. This phenomenon was also observed in different age groups from 300–550 days old when the suitable melatonin dose (10 mg) was used. A relatively high dose of implanted melatonin (30 mg) had a negative effect on the egg-laying rate. In addition to the increase in the egg-laying rate, melatonin (10 mg) implantation also improved the quality of the eggs, as indicated by the reduced sharpei egg rate.

Many studies have shown that melatonin plays an important role in animal reproduction. Melatonin could enhance the maturation of oocytes and the development of follicles in mammals^23^,^24^,^25^,^26^,^27^ and fish^28^,^29^. Despite the great differences between birds and mammals, melatonin may play a similar role in the maturation of oocytes and the development of follicles in birds as it does in mammals. Melatonin and its receptors were found to be present in the ovary. *MT1* and *MT2* were expressed in both the thecal and granulosa layers^30^. It was reported that MT1 and MT3 were negatively correlated with the total number of eggs yielded in hens 300 days of age that were exposed to different monochromatic light^31^. We observed that melatonin implantation did not influence the expression of ovarian *MT1* but up-regulated the expression of *MT2*. Interestingly, similar to *MT2, GnIHR* was also found to be expressed in both the thecal and granulosa layers^32^. It has been reported that E2 secretion and *GnIHR* expression regulate egg production and quality^33^,^34^,^35^,^36^. *GnIHR* is mainly expressed in the pituitary. It participates in the negative control of luteinizing hormone (LH) and follicle stimulating hormone (FSH)^21^. *GnIHR* was also found in the chicken ovary and is expressed in every stage of the follicle. Expression of *GnIHR* declines throughout sex maturation^32^. In the current study, melatonin was found to down-regulate expression of *GnIHR* in the ovary. This result indicated that melatonin-promoted ovulation in hens might be through the GnIHR-LH-FSH pathway.

Even though a positive correlation between GnIHR and MT2 expression in the testes of European starlings has been reported^22^, the concrete mechanism for this correlation is still not clear. Furthermore, their relationship in the ovary is virtually unknown. Granulosa cells are essential for oocyte maturation and ovarian ovulation. Glucose metabolism in mouse cumulus cells could prevent oocyte ageing^37^. Coculture with cumulus-derived cells *in vitro* promotes porcine and sheep oocyte maturation^38^. Melatonin and its receptor MT1 have been reported to be involved in the downstream reaction to luteinizing hormone and to participate in the regulation of luteinization in the mouse^39^. In the current study, it seems that the down-regulation of *GnIHR* expression by melatonin might be mediated by *MT2* in the granulosa layers and then promote ovulation.

Reactive oxygen species (ROS) caused by ageing and environmental stress will significantly affect an animal’s reproduction, suppressing the reproductive potential. The ROS caused by reproductive ageing is associated with changes in oocyte mitochondrial dynamics and function and mtDNA quantity and prevents oocyte maturation^40^. It has been reported that heat shock results in an inhibitory effect on porcine oocyte maturation *in vitro* by improving the ROS in the oocytes^41^. Maternal-restraint stress increases oocyte aneuploidy by impairing metaphase I spindle assembly and reducing spindle assembly checkpoint proteins in mice^42^. To combat the negative effects of the excessive ROS and promote the maturation of oocytes and ovulation, antioxidants are frequently used in the *in vitro* culture system^43^,^44^. Melatonin is a naturally occurring potent free radical scavenger and a broad-spectrum antioxidant^45^,^46^. As a result, the use of melatonin to prevent oxidative stress in cell culture or animal studies has been extensively reported^43^,^44^. It was reported that under stressful heat conditions, melatonin application improved the feeding efficiency of Japanese quail and reduced their oxidative stress^47^. Moreover, melatonin could enhance immunity in chickens against disease^14^,^48^,^49^. As a result, these activities of melatonin may also help the hens preserve their egg-laying rate after their egg-laying peak age.

A few studies have investigated the effects of melatonin on the quality of eggs. Some researchers have observed that melatonin supplementation reallocated the calcium distribution between bone and eggshell in laying hens, suggesting that melatonin could strengthen the bone and weaken the eggshell^50^. Other reports have shown that melatonin application increased the eggshell weight and thickness^51^. These findings are consistent with our observations that melatonin implantation significantly reduced the sharpei egg rate.

## Conclusion

Physiologically, once hens are older than 300 days of age, the hens have passed their egg-laying peak, and the egg-laying rate significantly declines with age^20^. Prolonging the egg-laying peak in chicken is task that remains to be accomplished. In the current study, we observed that implantation of the appropriate dose of melatonin (10 mg/hen) in laying hens up to 550 days of age significantly increased their egg-laying rate and egg quality. The results indicated that melatonin application may prolong the physiological egg-laying peak. Considering the low cost of melatonin, there may be an application for it in the poultry industry. The mechanistic studies revealed that melatonin implantation up-regulated the gene expression of *MT2* and down-regulated *GnIHR* in the granulosa cells of small white follicles. This finding indicated that melatonin may promote ovulation in hens through the GnIHR-LH-FSH pathway mediated by *MT2* activation.

## Materials and Methods

### Chemicals

The melatonin implants were made by the Specialty Institute, Chinese Academy of Agricultural Sciences.

### Animals

We selected 120 hens (Beijing Red No. 1) 300 days old, divided them into four groups, and treated them with either 0, 5, 10 or 20 mg of melatonin implants (C, 5 mg, 10 mg, and 20 mg groups, respectively). We randomly divided 240 hens (120 hens 360 days of age and 120 hens 470 days of age) into four groups at each age and treated them with 0, 10, 20 and 30 mg of melatonin (C, 10 mg, 20 mg, and 30 mg groups, respectively). We divided 60 hens 550 days old into 4 groups, and their treatment was the same as the hens in the 300-day-old groups. Hens of different ages came from different hatches.

Melatonin implants were implanted under the neck skin of the birds. Birds were reared under photostimulatory conditions (16 L:8D) with a corn-soybean meal diet of 110 g/hen/day. One cage (40 × 37 × 35 cm, length × width × height) contained 3 birds. The egg numbers and total egg weight of each group were monitored daily from at least 2 weeks before the treatment started until the termination of the study. All experimental procedures were approved by the animal care committee of the China Agricultural University, and all experiments were carried out in accordance with the relevant guidelines.

### Melatonin assay using high-performance liquid chromatography (HPLC)

To evaluate the melatonin level, blood samples (2 ml for each hen) were drawn from the wing sinus of the chicken. The sample preparation and detection were performed as described by Zhao *et al*.^52^.

### Analysis of progesterone (P4) and oestradiol-17β (E2) levels by radioimmunoassay

Blood samples (2 ml for each hen, n = 20) were drawn from the wing sinus of the chicken 3 months after melatonin implantation. Progesterone (P4) and oestradiol-17β (E2) were detected using radioimmunoassay using the method described by He *et al*.^53^.

### Gene expression assay using reverse-transcriptional PCR (real-time PCR)

The ovaries of hens 360 days of age (n = 10) were collected after 3 months of melatonin implantation and were immediately frozen in liquid nitrogen for future use. The total RNA extraction, reverse transcription PCR and quantitative real-time PCR were performed as described previously^54^. A housekeeping gene (β-actin) was used as the normalization control. The relative mRNA expression was calculated using the 2^−△△ct^ method. Primer sequences for the real-time PCR are listed in Table 2.

### Statistical analyses

The data were expressed as the means ± SEM. The statistical significance was analysed using an ANOVA followed by the Dunnett’s t-test. The significance level was set at p < 0.05.

## Additional Information

**How to cite this article**: Jia, Y. *et al*. Melatonin implantation improved the egg-laying rate and quality in hens past their peak egg-laying age. *Sci. Rep.* **6**, 39799; doi: 10.1038/srep39799 (2016).

**Publisher's note:** Springer Nature remains neutral with regard to jurisdictional claims in published maps and institutional affiliations.

## Figures and Tables

**Table 1:** Effect of melatonin implantation on blood E2 and P4 levels.

	Implantation age	Time/month	10 mg	control
E2 (pg/ml)	360 d	0	—	—
		3	900.1 ± 34.4^a^	780.0 ± 44.6^b^
	470 d	0	—	—
		3	823.3 ± 80.4	828.0 ± 83.2
P4 (pg/ml)	360 d	0	—	—
		3	116.3 ± 18.1	92.7 ± 10.5
	470 d	0	115.3 ± 10.5	233.9 ± 80.3
		3	72.1 ± 8.2	97.0 ± 15.2

In the same line, different small letters mean significance of p < 0.05. Data were expressed as the means ± SEM.

**Table 2:** Primers used in this study.

Primer	GenBank ID	Production length bp	Sequence
β-actin-F	L08165	154	GAGAAATTGTGCGTGACATCAAGG
β-actin-R			CACCTGAACCTCTCATTGCCA
LHR-F	u31987	193	CTCAGGCGGATACACAACGA
LHR-R			TCAGAACGGCTTCCAGCAGG
FSHR-F	NM_205079.1	191	TACCCGTCGTCCATAAGGTGC
FSHR-R			GCTCATCCAGGCAGGTTCCATT
ERα-F	NM—205183.2	157	TATTGATGATCGGCTTAGTCTGGCG
ERα-R			CGAGCAGCAGTAGCCAGTAGCA
GnIHR-F	AB193127	139	CACTGATGCTGCTGACAGACTAC
GnIHR-R			CTCATTGAAGTAGCCGTAGATGATGG
MT1-F	NM_001097538.1	127	CAGGACTGCCCTTGTGCC
MT1-R			CACACTTGGCACATCCTGC
MT2-F	NM_205275.1	99	AACCGACCCGAACTGAACCA
MT2-R			CAGCGGCAGTTCTTGCACTT
SOD2-F	NM_204211.1	157	CGCAAGGCAGAAGCACACTC
SOD2-R			CAGCGCCTCTTTGTATTTCTCC
